# Single-cell RNA sequencing reveals compartmental remodeling of tumor-infiltrating immune cells induced by anti-CD47 targeting in pancreatic cancer

**DOI:** 10.1186/s13045-019-0822-6

**Published:** 2019-11-27

**Authors:** Yu Pan, Fengchun Lu, Qinglin Fei, Xingxing Yu, Ping Xiong, Xunbin Yu, Yuan Dang, Zelin Hou, Wenji Lin, Xianchao Lin, Zheyang Zhang, Minggui Pan, Heguang Huang

**Affiliations:** 1 Department of General Surgery, Fujian Medical University Union Hospital, No. 29 Xinquan Road, Fuzhou, 350001 China; 2 Department of Obstetrics and Gynecology, 900 Hospital of the Joint Logistics Team, 156 North Xi-er Huan Road, Fuzhou, 350001 China; 3 Department of Pathology, Fujian provincial hospital, Fuzhou, 350001 China; 4 Department of Comparative medicine, 900 Hospital of the Joint Logistics Team (Dongfang Hospital), Xiamen University Medical College, 156 North Xi-er Huan Road, Fuzhou, 350001 China; 5 Department of Radiology, Quanzhou First Hospital of Fujian Medical University, Quanzhou, 362000 China; 6 College of Bioinformatics Science and Technology, Harbin Medical University, Harbin, 150086 China; 7 Department of Oncology and Hematology and Division of Research, Kaiser Permanente, Santa Clara, CA 95051 USA

**Keywords:** CD47, PD-L1, Immunotherapy, Pancreatic cancer, Immune checkpoint inhibitor

## Abstract

**Background:**

Human pancreatic ductal adenocarcinoma (PDAC) responds poorly to immune checkpoint inhibitor (ICPi). While the mechanism is not completely clear, it has been recognized that tumor microenvironment (TME) plays key roles. We investigated if targeting CD47 with a monoclonal antibody could enhance the response of PDAC to ICPi by altering the TME.

**Methods:**

Using immunohistochemistry, we examined tumor-infiltrating CD68^+^ pan-macrophages (CD68^+^ M) and CD163^+^ M2 macrophages (CD163^+^ M2) and tumor expression of CD47 and PD-L1 proteins in 106 cases of PDAC. The efficacy of CD47 blockade was examined in xenograft models. CD45^+^ immune cells from syngeneic tumor models were subjected to single-cell RNA-sequencing (scRNA-seq) by using the 10x Genomics pipeline.

**Results:**

We found that CD47 expression correlated with the level of CD68^+^ M but not CD163^+^ M2. High levels of tumor-infiltrating CD68^+^ M, CD163^+^ M2, and CD47 expression were significantly associated with worse survival. CD47^high^/CD68^+^ M^high^ and CD47^high^/CD163^+^ M2^high^ correlated significantly with shorter survival, whereas CD47^low^/CD68^+^ M^low^ and CD47^low^/CD163^+^ M2^low^ correlated with longer survival. Intriguingly, CD47 blockade decreased the tumor burden in the Panc02 but not in the MPC-83 syngeneic mouse model. Using scRNA-seq, we showed that anti-CD47 treatment significantly remodeled the intratumoral lymphocyte and macrophage compartments in Panc02 tumor-bearing mice by increasing the pro-inflammatory macrophages that exhibit anti-tumor function, while reducing the anti-inflammatory macrophages. Moreover, CD47 blockade not only increased the number of intratumoral CD8^+^ T cells, but also remodeled the T cell cluster toward a more activated one. Further, combination therapy targeting both CD47 and PD-L1 resulted in synergistic inhibition of PDAC growth in the MPC-83 but not in Panc02 model. MPC-83 but not Panc02 mice treated with both anti-CD47 and anti-PD-L1 showed increased number of PD-1^+^CD8^+^ T cells and enhanced expression of key immune activating genes.

**Conclusion:**

Our data indicate that CD47 targeting induces compartmental remodeling of tumor-infiltrating immune cells of the TME in PDAC. Different PDAC mouse models exhibited differential response to the anti-CD47 and anti-PD-L1 blockade due to the differential effect of this combination treatment on the infiltrating immune cells and key immune activating genes in the TME established by the different PDAC cell lines.

## Introduction

Pancreatic ductal adenocarcinoma (PDAC) is a highly aggressive malignancy with 5-year survival rate of approximately 9% [1]. Immune checkpoint inhibitors (ICPis) have shown little activity in PDAC despite their broad efficacy in many other malignancies [2–5], likely related to the nature of the tumor microenvironment (TME) in PDAC [6, 7]. Some previous studies [8–10] showed that PDAC TME often contained a wide range of CD4^+^ T cells, CD8^+^ T cells, regulatory T cells, neutrophils, and macrophages infiltration.

Tumor-associated macrophages (TAM) are the most abundant tumor-infiltrating immune cells in PDAC [11]. They can be divided into two subsets: immune-stimulatory macrophages (or M1 macrophages, M1) and immune-regulatory macrophages (or M2 macrophages, M2). M1 secretes gamma interferon (IFN-훾) and other inflammatory cytokines, whereas M2 produces immunosuppressive cytokines such as interleukin 10 (IL-10) that participates in the tumor immune escape in the TME and promotes tumor cell proliferation [12, 13]. Moreover, TAM was associated with poor survival in patients with PDAC [10]; thus, TAM may be a valid therapeutic target of PDAC. Some recent studies [14–16] showed that CD47, a “don’t eat me” signal that binds to its receptor signal regulatory protein α (SIRPα) on phagocytes to suppress macrophage phagocytosis, was widely expressed on the surface of malignant cells. Evidence has accumulated that anti-CD47 targeting can induce macrophage phagocytosis of tumor cells and may improve cell-mediated immune response [11, 16, 17]. Blocking CD47-SIRPα pathway has been shown to be effective in inhibiting several malignancies in preclinical studies [15, 18]. However, the expression of CD47 in PDAC has not been comprehensively studied. Also, the relationship between the tumor expression of CD47 and TAM in PDAC remains unclear. The impact of CD47 blockade on macrophages, CD4- and CD8-positive T cells is not understood.

In this study, we explored the effect of targeting CD47 on the TME of PDAC and if targeting both CD47 and PD-L1 could enhance the inhibitory effect on PDAC growth. We investigated the effect of anti-CD47 in patient-derived PDAC xenografts and studied the mechanism of such effect using single-cell RNA-sequencing (scRNA-seq), a high dimensional profiling to evaluate functional and genetic changes of tumor-infiltrating immune cell populations of syngeneic mouse models following CD47 targeting.

## Materials and methods

### Patients and tissue samples

Human pancreatic cancer tumor samples were collected from the patients who received surgery at Fujian Medical University Union Hospital, Fuzhou, China, from November 2010 to January 2019. All patients had histologically confirmed PDAC. Patients with neoadjuvant treatment, inflammatory diseases, or active infection were excluded. A total of 106 patients who had been diagnosed with PDAC were enrolled in the study. The stage of each patient was assessed based on the American Joint Committee on Cancer version 8 (AJCC 8). Informed consent was obtained before sample collection. The study was approved by the Committee for the Ethical Review of Research, Fujian Medical University Union Hospital. Formalin-fixed paraffin-embedded samples were obtained for immunohistochemistry analysis.

### Cell lines

The murine PDAC cell lines Panc02 and MPC-83, syngeneic to C57BL/6 mice, and Kunming (KM) mice were obtained from Shanghai Aolu Biological Technology Co. Ltd (Shanghai, China). Human pancreatic cancer cell lines including PANC-1, BxPC-3, SW1990, CFPAC-1, and AsPC-1 were obtained from the Cell Bank, Chinese Academy of Sciences (Shanghai, China). All cell lines were genotyped for identification by the Cell Bank, Chinese Academy of Sciences, and were tested to rule out mycoplasma contamination.

### Mice

Male athymic nude (BALB/c-nu) mice, 4–5 weeks of age, male C57BL/6 mice, 5 weeks of age, and male KM mice, 5 weeks of age, were obtained from Beijing Vital River Laboratory Animal Technology Co., Ltd. (Beijing, China). Male NCG (NOD-Prkdc^em26Cd52^Il2rg^em26Cd22^/NjuCrl) mice, 4–5 weeks of age, were obtained from Nanjing Biomedical Research Institute of Nanjing University (Nanjing, China).

### Antibodies

Monoclonal rabbit anti-human PD-L1 antibody (E1L3N, #13684) and monoclonal rabbit anti-human CD68 antibody (D4B9C, #76437) were obtained from Cell Signaling Technology and the polyclonal rabbit anti-human/mouse CD47 antibody (ab175388), monoclonal rabbit anti-human/mouse CD163 antibody (clone EPR19518), monoclonal rabbit anti-mouse PD-L1 antibody (clone EPR20529), rabbit anti-CD4 antibody (EPR19514), anti-CD8 antibody (YTS169.4), rabbit anti-iNOS antibody (ab15323), and rabbit Anti-CD206 antibody (ab64693) were from Abcam. Anti-mouse CD8a monoclonal antibody, PE (Clone: 53-6.7) were purchased from eBioscience. Anti-mouse CD279 (PD-1), FITC (Clone: 29F.1A12) were purchased from Biolegend.

### In vivo tumorigenicity assay

Patient-derived xenograft (PDX) model was performed according to the previous studies [19, 20]. PDAC tumor samples P962 and P989 were collected from fresh human surgical specimens at Fujian Medical University Union Hospital. Tumors were placed in RPMI supplemented with 10% fetal bovine serum (FBS) and cut into 0.3 × 0.3 × 0.3 cm pieces. The right axilla of each nude mice or NCG mice was sterilized and a small incision on the right axilla create a subcutaneous pocket, and then the 0.3 × 0.3 × 0.3 cm tumor piece was inserted into the pocket (P1 generation). When tumors reach 1000 mm^3^, the mice were sacrificed and tumors were removed and passed to a secondary colony of mice (P2 generation). We implanted 20 tumors in 10 nude mice and 10 NCG mice, respectively. Four weeks after tumor implantation, mice were divided into two groups (*n* = 5 tumors per group): control, or an anti-human CD47 in vivo mAb (200 μg/day i.p., Clone No. B6.H12, BioXcell), for 2 weeks. After treatment, mice were sacrificed and tumors were removed and weighed.

The syngeneic tumor model was established in accordance with our previously described protocol [21]. Panc02 cells or MPC-83 cells were subcutaneously implanted into 20 C57BL/6 mice or 20 KM mice. When the tumor reached 100 mm^3^, tumor-bearing mice were randomly divided into four groups. Then, tumor-bearing mice were treated with mouse IgG (200 μg/day i.p., Clone No. MPC-11, BioXcell), an anti-mouse CD47 in vivo mAb (200 μg/day i.p., Clone No. MIAP301, BioXcell), an anti-mouse PD-L1 in vivo mAb (mAb; 200 μg/day i.p., Clone No. 10F.9G2, BioXcell), or anti-CD47 mAb + anti-PD-L1 mAb. After 2 weeks of treatment, mice were sacrificed, and tumors were removed and weighed. All experiments were approved by the Ethics Committee for Animal Research of 900 Hospital of the Joint Logistics Team.

### Tissue digestion

Complete media was prepared with RPMI-1640 (Hyclone), 10% FBS (Gibco), and 1% penicillin-streptomycin (Hyclone). Tumor tissues from mouse xenograft models were each minced with scissors and enzymatically digested in complete media supplemented with 1.0 mg/ml collagenase type IV (Sigma), 30 U/ml DNase type I (Sigma), and 0.5 mg/ml HAase type V (Sigma) for 50 min at 37 °C. Then the cells were filtered through the 70 μm cell strainers (Miltenyi Biotec), washed with phosphate-buffered saline (PBS), lysed in red blood cell buffer (BioTeke, China), and resuspended in PBS. Tumor-infiltrating immune cells (CD45^+^ cells) were sorted by mouse TIL (CD45) MicroBeads (Miltenyi Biotec) according to the manufacturer’s protocol.

### Peripheral blood mononuclear cell isolation

The peripheral blood mononuclear cells (PBMCs) were isolated from xenograft mouse models by Ficoll-Hypaque gradient centrifugation (Haoyang Biotech, Tianjin, China).

### Isolation of splenocytes

The spleen was removed from xenograft mouse models, placed in the sterile plastic dish with PBS, and then minced and ground on the 70 μm cell strainers, dispersing into a single-cell suspension. Cells were washed with PBS, lysed in red blood cell buffer, and resuspended in PBS.

### Flow cytometry analysis

To determine the proportion of PD-1^+^CD8^+^ T cells in lymphocytes, the cells from the tumor, spleen, and peripheral blood of mouse syngeneic tumor models were stained with PD-1-FITC mAb and CD8a-PE mAb and performed on the BD Accuri C6 flow cytometer (BD Biosciences) as previously described [22].

### Immunoblotting

Western blotting for PD-L1 and CD47 in pancreatic cancer cells was performed using methods described previously [21].

### Immunohistochemistry (IHC)

Immunohistochemical analysis and PD-L1 status were defined as our previously described protocol [21]. The CD47 protein staining intensity was assessed based on the intensity score of 0 to 3 scale with 0 for negative expression, 1 to indicate weak, 2 to indicate moderate, or 3 to indicate strong. The percentage of tumor cells stained positive was assessed based on the score of 1 to 3 representing < 30%, 30–80%, and > 80% cells. The CD47 protein expression was defined as high if the score is ≥ 4. Five areas of a representative field were counted at × 400 magnification for CD68^+^ and CD163^+^ macrophages, and the average was calculated. High infiltration of CD68^+^ macrophages was defined as more than 200 positive cells on average, whereas that of CD163^+^ macrophages was defined as more than 100 positive cells, as described previously [12]. All specimens were evaluated by two pathologists who were blinded to the patients’ clinical information.

### Immunofluorescence

Immunofluorescence assays were performed to identify the location of PD-L1 and CD47 in pancreatic cancer cells, as previously described [21].

### Single-Cell RNA sequencing

#### Cell capture and cDNA synthesis

Using single-cell 5′ Library and Gel Bead Kit (10x Genomics, 1000006) and Chromium Single Cell A Chip Kit (10x Genomics, 120236), the cell suspension (300–600 living cells per microliter determined by Count Star) was loaded onto the Chromium single cell controller (10x Genomics) to generate single-cell gel beads in the emulsion according to the manufacturer’s protocol. In short, single cells were suspended in PBS containing 0.04% BSA. Then the cells were added to each channel, and the target cell will be recovered. Captured cells were lysed and the released RNA were barcoded through reverse transcription in individual GEMs [23]. Reverse transcription was performed on a S1000TM Touch Thermal Cycler (Bio Rad) at 53 °C for 45 min, followed by 85 °C for 5 min, and hold at 4 °C. The cDNA was generated and then amplified, and quality assessed using an Agilent 4200 (performed by CapitalBio, Beijing).

#### Single-cell RNA-Seq library preparation

According to the manufacture’s introduction, single-cell RNA-seq libraries were constructed using Single Cell 5′ Library and Gel Bead Kit. The libraries were sequenced using an Illumina Novaseq6000 sequencer with a sequencing depth of at least 77,618 reads per cell with pair-end 150 bp (PE150) reading strategy (performed by CapitalBio, Beijing).

### Data preprocessing with Cell Ranger pipeline

The Cell Ranger software was obtained from 10x Genomics website https://support.10xgenomics.com/single-cell-gene-expression/software/downloads/latest. Alignment, filtering, barcode counting, and UMI counting were performed with Cell Ranger count module to generate feature-barcode matrix and determine clusters.

### Data preprocessing with Seurat package

The Seurat pipeline was applied to the data [24, 25]. Genes expressed in less than three cells and cell expressed less than 400 and more than 5000 genes were excluded. The data were normalized and the scale factor was 104. Most variable genes were detected by the FindVariableFeatures function and used for subsequent analysis. Principle component analysis (PCA) was performed on about 3000 genes with PCA function. The first 40 PCA components were used for the tSNE dimension reduction of the scaling matrix (with only the majority of variable genes) to obtain a two-dimensional representation of the cell state. For clustering, we used FindClusters function, which realized the modular and optimized clustering algorithm of SNN (shared nearest neighbor) based on 40 PCA components, and its resolution was 0.5–1, resulting in 19–25 clusters. A resolution of 0.6 was selected for analysis.

### Cluster specific gene identification and marker-based classification

To confirm marker genes, the function of FindAllMarkers was combined with likelihood-ratio test of single cell gene expression. For each cluster, only the genes expressed in more than 25% of the cells with at least 0.25-fold difference were considered. To represent clusters, ImmGen and Enrichr were used. For pathway analysis, intra clusters (e.g., T cells, macrophages) with different parameters (zerofold and at least 10% of the cell threshold to express this gene in clusters) were compared. To represent heatmap, the average expression of the markers within each cluster was used.

### Lymphoid clusters analysis

To detect lymphocytes, clusters expressing Cd3e were extracted from the collected samples. Most variable genes, PCA, tSNE, clustering, and marker selection analysis were performed as described before [24].

### Enrichment analysis

GO enrichment and KEGG enrichment of cluster markers were performed using KOBAS software with Benjamini-Hochberg multiple testing adjustment, using the top 20 markers gene of cluster. The results were visualized using R package.

### Bulk RNA-seq data processing

The bulk RNA-seq data were processed using the same Seurat pipeline as a single cell RNA-seq data.

### Statistical analysis

Quantitative data were expressed as the mean ± standard deviation (SD) and analyzed based on variance and Student’s *t* tests. Chi-square tests were performed to compare PD-L1, CD47, CD68, and CD163 and clinical features. Spearman’s rank correlation was evaluated to determine the correlation between CD47, CD68, and CD163. OS was measured from the day of death from any cause or the last censored follow-up. Survival and date of diagnosis analysis methods were similar to those previously described [21]. Data were analyzed using the Statistical Package for Social Science version 22.0 (SPSS, IBM, Armonk, USA).

## Results

### Patient characteristics

Additional file 1: Table S1 shows the clinico-pathological characteristics of 106 patients with PDAC. The median age of patients was 61 years (35–82). Fifty-eight percent of patients were males, and 79.3% of patients had TNM stage II (45 cases) and III (39 cases) disease. Neoadjuvant therapy was not given to any of the patients. Median overall survival (OS) was 12.1 months.

### TAM, expression of CD47, and PD-L1 in human PDAC

**Fig. 1:**
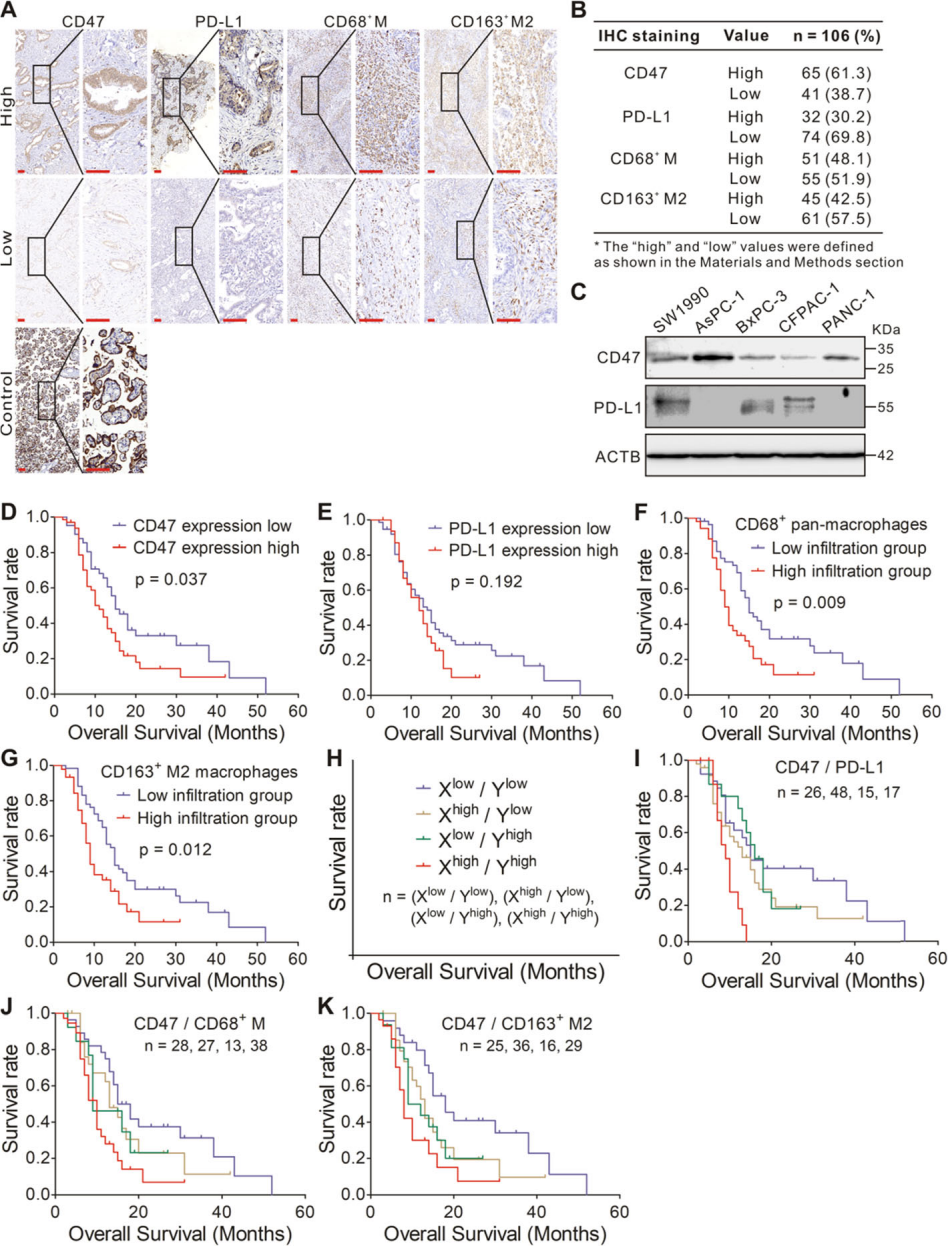
Immunostaining of CD47, PD-L1, CD68, and CD163 in human PDAC. **a** Staining with an anti-CD47, anti-PD-L1, anti-CD68, and anti-CD163 antibody in the human PDAC tissue samples at × 100 magnification and × 400 magnification. Scale bar = 50 μm (red line at the bottom left). **b** Results of immunohistochemical staining. **c** Immunoblotting of CD47 and PD-L1 in PDAC cell lines. ACTB (β-actin) was used as a normalization control. **d** Kaplan–Meyer plot of OS in 106 PDAC patients with high or low tumor CD47 expression. **e** Kaplan–Meyer plot of OS in 106 PDAC patients with high or low tumor PD-L1 expression. **f**, **g** Kaplan–Meyer plot of OS in 106 PDAC patients with high or low tumor-infiltrating CD68^+^ (**f**) or CD163^+^ macrophages (**g**). **h**–**k** Kaplan–Meyer plot of OS among four groups of patients divided on the combinations of two variables with high and low expression. **h** is an illustration of **i**–**k**. The four groups are *X*^low^ and *Y*^low^ in blue, *X*^high^ and *Y*^low^ in gold, *X*^low^ and *Y*^high^ in green, and *X*^high^ and *Y*^high^ in red. “*X*” and “*Y*” represent the two different variables (CD47, PD-L1, CD68^+^ M, or CD163^+^ M2). “*n*” represent the numbers of patients in four groups (blue, gold, green, and red)

To understand the relationship among CD47, PD-L1, and TAM in PDAC, we stained the tumor specimen from 106 PDAC patients with anti-CD47, anti-PD-L1, anti-CD68, and anti-CD163 antibodies. CD47 expression in human placenta was used as positive control (Fig. 1a). The representative IHC staining of CD47, PD-L1, CD68, and CD163 were shown in Fig. 1a. We used the antigen CD68 for pan-macrophages (CD68^+^ M) and CD163 for M2 macrophages (CD163^+^ M2). IHC staining showed that CD47 and PD-L1 was highly expressed in 61.3% and 30.2% of PDAC tissues (Fig. 1b). We next investigated the expression of CD47 and PD-L1 in five human PDAC cell lines by using Western blotting. CD47 was expressed at various levels in all five cell lines (Fig. 1c), and three cell lines (SW1990, BxPC-3, and CFPAC-1) showed PD-L1 expression, similar to our previous study [21].

In 48.1% and 42.5% of the cases, high CD68^+^ and high CD163^+^ macrophage populations were detected (Fig. 1b). We found that 74.5% of higher numbers of CD68^+^ M were high CD163^+^ M2. CD68^+^ M and CD163^+^ M2 showed a significant positive correlation with each other by using case-by-case analysis (*r* = 0.625, *p* < 0.001; Additional file 1: Table S1). Moreover, CD68^+^ M closely correlated with CD47 expression (*r* = 0.261, *p* = 0.007; Additional file 1: Table S1), but no significant correlation was found between CD163^+^ M2 and CD47 expression (*r* = 0.055, *p* = 0.571; Additional file 1: Table S1). Compared to low CD163^+^ macrophage populations, the high CD163^+^ counts were associated with higher pT-stage (*p* = 0.015; Additional file 1: Table S1) and the trend towards larger tumor diameter (*p* = 0.058; Additional file 1: Table S1). In contrast, CD68^+^ M and CD47 expression did not correlate with most of the clinico-pathological variables, such as histological grade, clinical stage, tumor diameter, vascular invasion, and postoperative chemotherapy.

### TAM and tumor expression of CD47 correlated with poor outcome in PDAC patients

Univariate analysis showed that the variables associated with OS included tumor diameter [hazard ratio (HR) = 1.643; *p* = 0.038], pN-stage (HR = 1.82; *p* < 0.001), and grade (HR = 2.478; *p* = 0.001; Additional file 1: Table S2). Patients with high tumor expression of CD47 had worse OS (HR = 1.673; *p* = 0.037; Table 2; Fig. 1d), compared to those with low CD47 expression. Similar to our previous study [21], PD-L1 expression was not significantly associated with OS (Fig. 1e). Moreover, high numbers of CD68^+^ M and CD163^+^ M2 cells within the tumor were significantly associated with worse OS (HR = 1.892, 1.845; *p* = 0.009, 0.012; Additional file 1: Table S2; Fig. 1f, g). We also performed multivariate analysis to determine if CD47 expression or TAM remains independent predictors of OS. The variables of CD47 expression, CD68^+^ M or CD163^+^ M2, tumor diameter, N stage, and grade were included in the multivariate analysis. We found that tumor CD47 expression (HR = 1.703; *p* = 0.038), CD68^+^ M (HR = 1.853; *p* = 0.012), CD163^+^ M2 (HR = 1.898; *p* = 0.014), tumor diameter (HR = 1.626; *p* = 0.047), grade (HR = 1.745; *p* = 0.011), and N stage (HR = 1.831; *p* < 0.001) were independent factors associated with OS (Additional file 1: Table S2).

To further determine the prognostic value of CD47 expression and TAM, we examined the effect of these immune biomarkers on the OS of PDAC patients (Fig. 1h). We found that patients whose tumor cells had high expression of CD47 and PD-L1 (CD47^high^ /PD-L1^high^) were associated with worse OS compared to low CD47 and PD-L1 expression (CD47^low^/PD-L1^low^) (*p* = 0.003, Fig. 1i). Patients whose tumor had CD47^high^ and high tumor-infiltrating CD68^+^ M (CD68^+^ M^high^) and the patients whose tumor had CD47^high^ and high tumor-infiltrating CD163^+^ M2 (CD163^+^ M2^high^) were associated with worse OS (*p* = 0.003, Fig. 1j; *p* = 0.005, Fig. 1k), compared to patients with CD47^low^ and to patients with low tumor-infiltrating CD68^+^ M (CD68^+^ M^low^), and CD47^low^ and low tumor-infiltrating CD163^+^ M2 (CD163^+^ M2^low^) (*p* = 0.018, Fig. 1j; *p* = 0.007, Fig. 1k). By multivariate analysis with the variables including tumor diameter, TNM stage, and grade, we found that CD47^high^/CD68^+^ M^high^ (HR = 2.126; *p* = 0.006), CD47^high^/CD163^+^ M2^high^ (HR = 1.873; *p* = 0.035), CD47^low^/CD68^+^ M^low^ (HR = 0.47; *p* = 0.01), and CD47^low^/CD163^+^ M2^low^ (HR = 0.376; *p* = 0.002) were independent prognostic factors for OS (Additional file 1: Table S3). These results reveal that the combination of different immune markers may be of predictive value for OS in patients with PDAC.

### The effect of anti-CD47 targeting in PDAC mouse models

**Fig. 2:**
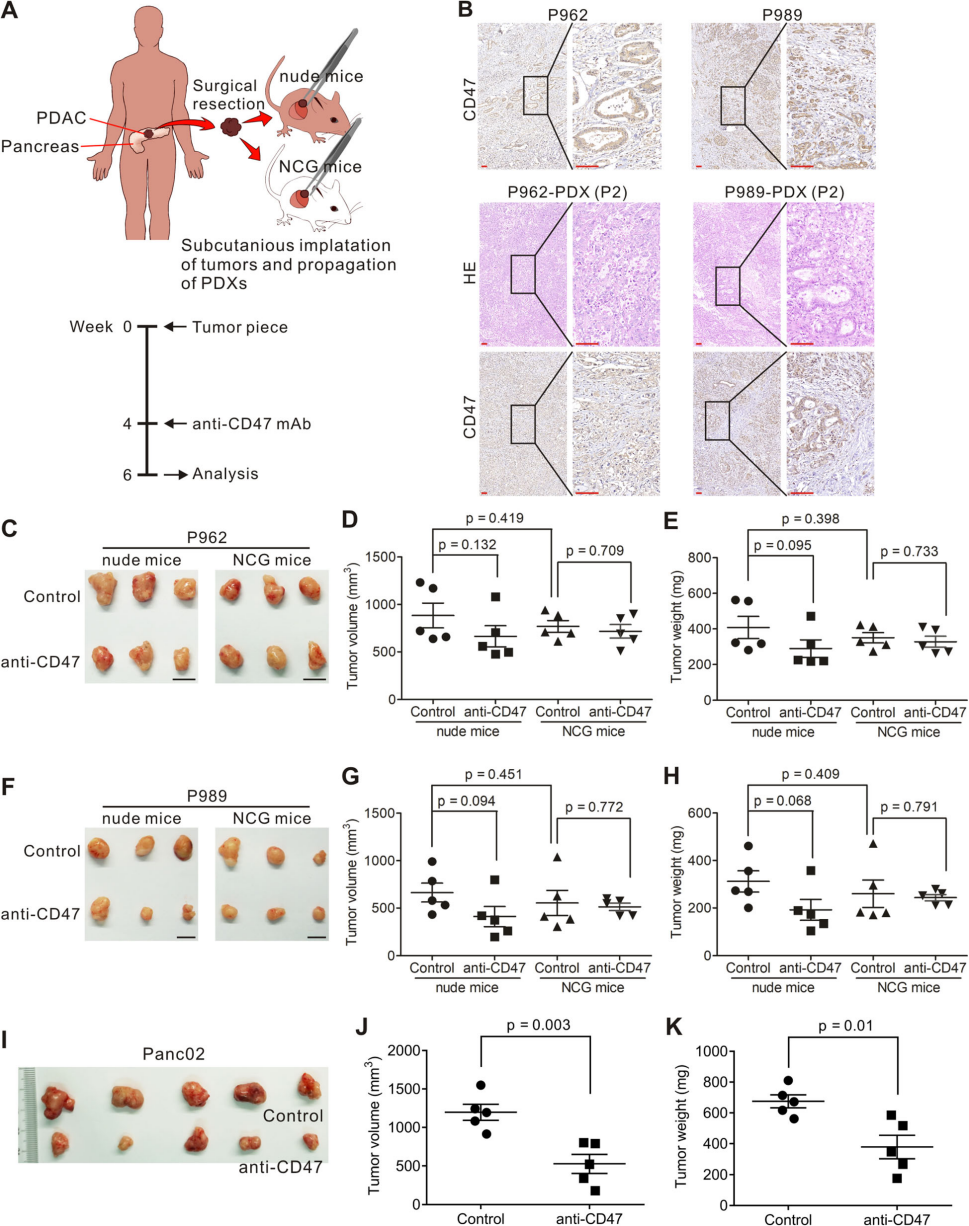
Effect of CD47 blockade on the patient-derived PDAC xenograft models. **a** Schematic of patient-derived PDAC xenograft establishment and tumor immunotherapy design. **b** Staining with an anti-CD47 antibody in two human PDAC tissue samples P962 and P989 at × 100 magnification and × 400 magnification (upper panels). H&E and anti-CD47 antibody staining of tumor tissues from P962 and P989 patient-derived xenograft models (P2 generation) at × 100 magnification and × 400 magnification (lower panels). Scale bar = 50 μm (red line at the bottom left). **c**–**h** P962 and P989 xenografts from nude and NCG mice were treated intraperitoneally with IgG control or anti-CD47 mAb at 200 μg/day for 2 weeks. Each group contained five animals. Tumor volume and weight were then measured (**d**, **e**, **g**, and **h**). (**i**–**k**) Panc02 cells were transplanted to C57BL/6 mice. When the tumor reached 100 mm^3^, tumor-bearing mice were treated with anti-CD47 antibodies for 14 days. Tumor volume and weight were then measured (**j**, **k**)

To examine the effect of anti-CD47 mAb on PDAC, we used the tumors from two patients with PDAC (P962 and P989) to create tumor implantations in nude mice and in NCG mice. NCG mice lack cell-mediated immunity and do not produce cytokine production and have no functional B cells, macrophages, and NK cells [26]. The mouse models were established and treated as shown in Fig. 2a. The expression of CD47 in parental tumor and xenograft was confirmed by IHC (Fig. 2b). After 2 weeks of anti-CD47 treatment, mice were sacrificed, and tumors were removed and weighed. For both P962 and P989, nude mice had the similar tumor burden when compare with NCG mice, as assessed by tumor volume (*p* = 0.419, 0.451) and weight (*p* = 0.398, 0.409; Fig. 2c–h). P962 and P989 nude mice treated with anti-CD47 mAb had reduced tumor burden (Fig. 2c–h). However, in NCG mouse models using the same human tumor implantation, treatment with anti-CD47 did not reduce tumor growth (Fig. 2c–h). This is likely related the immunodeficiency of the NCG mice that lacks functional T cells and innate immunity response [27, 28].

We investigated if the anti-tumor effect of CD47 targeting requires both innate and adaptive immunity, by implanting Panc02 cells to C57BL/6 mice. Using this mouse model, we found that treatment with anti-CD47 alone resulted in significantly reduced tumor growth compared to the untreated animals (Fig. 2i–k). These data suggested that an intact immune system may be required for effective CD47 targeting to establish the immunotherapeutic effect.

### Composition of tumor-infiltrating immune cell subpopulations identified by single-cell RNA-seq

**Fig. 3:**
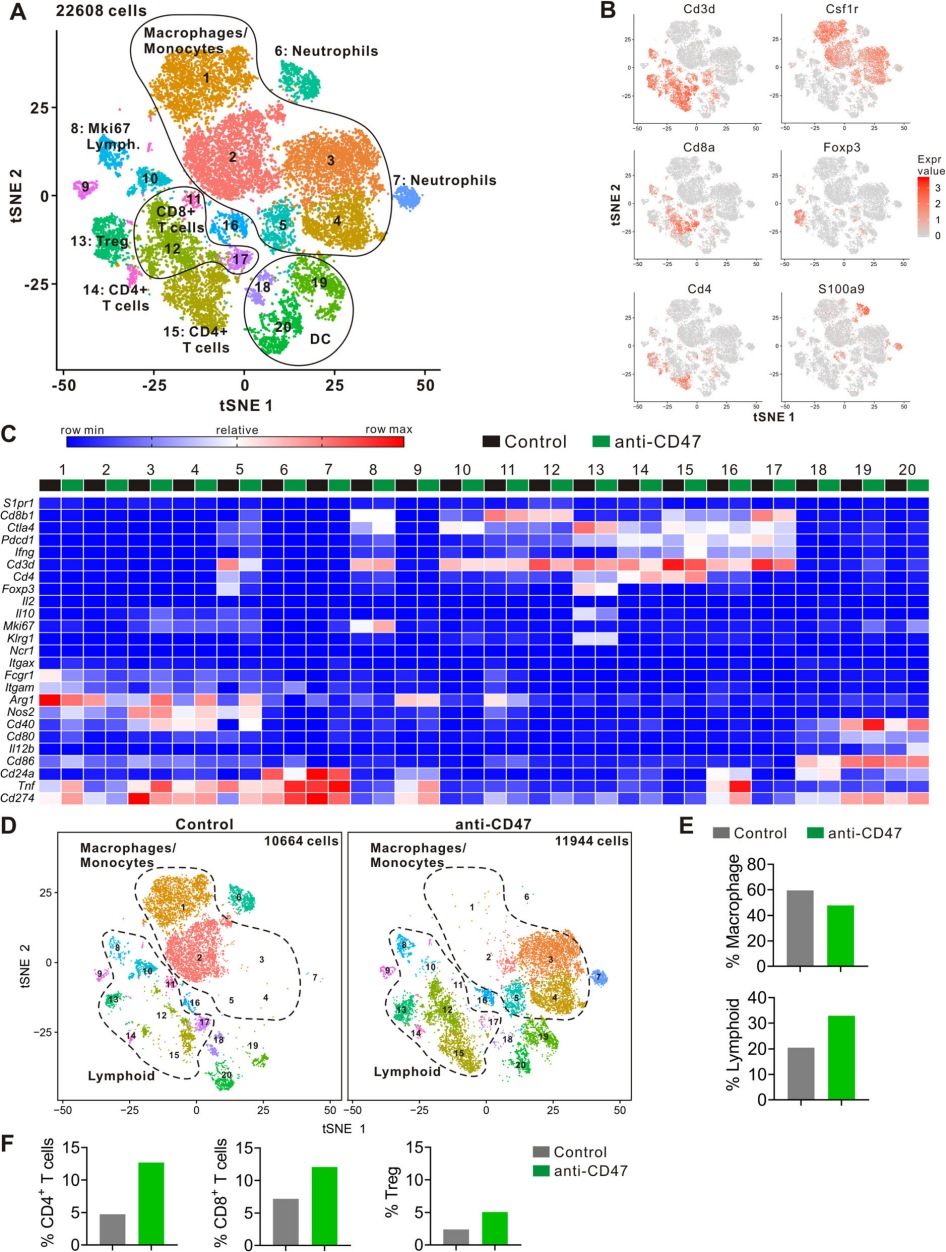
Identification of intratumoral immune cell clusters by scRNA-seq. **a** tSNE plot of intratumoral immune cells from two groups merged. **b** tSNE plot of immune cells displaying marker gene expression. **c** Heatmap displaying expression of select genes in each cell subpopulation. **d** tSNE plots with annotated clusters of intratumoral immune cells. **e**–**f** Proportion of cells in individual subpopulation by condition

To further understand the mouse immune cell subpopulations associated with anti-tumor response following anti-CD47 treatment, we harvested the tumors on day 15 following the anti-CD47 treatment and analyzed the CD45-positive immune cells by scRNA-seq with 10x Genomics pipeline (Additional file 1: Figure S1A and B). To better define the subpopulation structure of the tumor-infiltrating immune cells, we computationally pooled the data from the control and the anti-CD47 group representing a total of 22,608 cells. We used graph-based clustering to identify transcriptional clusters consisting of individual cell types (Fig. 3a). Comparison with the ImmGen database and assessment of known cell-type markers resulted in elaboration of eight lymphoid clusters, five monocyte/macrophage clusters, three neutrophil clusters, and three dendritic cell (DC) clusters (Fig. 3a–c). Following anti-CD47 treatment, the proportion of monocyte/macrophage populations were decreased, whereas the lymphoid populations were increased, including the proportions of CD4^+^ T cells, CD8^+^ T cells, and regulatory T cells (Tregs) (Fig. 3d–f). Using Immunohistochemical staining, we also found that the number and percent of CD4^+^ T cells and CD8^+^ T cells were improved after anti-CD47 treatment (Additional file 1: Figure S2).

**Fig. 4:**
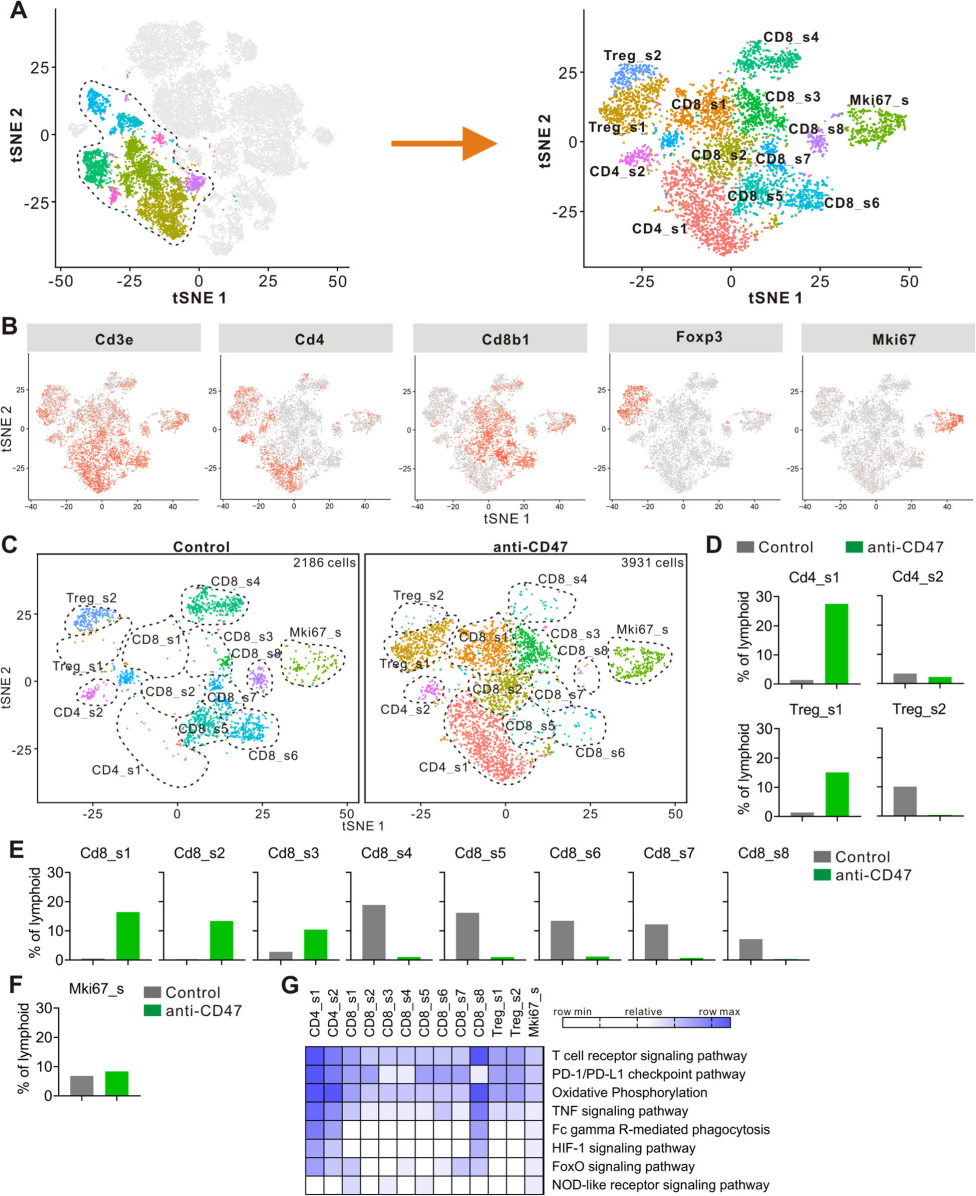
Anti-CD47 treatment remodels intratumoral lymphoid cells. **a** tSNE plot from merged data of intratumoral lymphoid cells. **b** tSNE plot of lymphoid cells displaying select marker-gene expression. **c** tSNE plots with annotated clusters of intratumoral lymphoid cells. **d**–**f** Proportion of cells in individual subpopulation by condition. **g** Heatmap of KEGG identifying pathway enrichment by each subpopulation

To better understand and more accurately define the lymphoid clusters identified by single-cell RNA-seq, we computationally separated lymphoid cells (6117 cells total for two groups) and reanalyzed the data (Fig. 4a). This approach produced 13 distinct lymphoid clusters broadly defined by the distribution of classical marker genes (Fig. 4b and Additional file 1: Figure S3). Clusters are named as “XXX_s#”, where “XXX” represents the cell type, “s” represents the scRNA-seq, and “#” represents the different cluster.

#### Changes in CD4^+^ T cells

Single-cell RNA-seq revealed two distinct clusters of FoxP3^−^ CD4^+^ T cells (CD4_s1 and CD4_s2) (Fig. 4c). CD4_s1 and CD4_s2 expressed high levels of *Cd4* and functional markers such as *Lag3*, *Pdcd1* (PD-1), *Ctla4*, and *Icos* (Fig. 4b and Additional file 1: Figure S3). CD4_s2 was distinguishable from CD4_s1 by its higher expression of *CD44*, *Cd200*, and *Ccr7*, and lower expression of *Ccl5* and *Lag3* (Additional file 1: Figure S3). KEGG revealed that both CD4_s1 and CD4_s2 displayed the pathways associated with oxidative phosphorylation and Fc gamma R-mediated phagocytosis, and the signaling via T cell receptor, PD-1/PD-L1 checkpoint, TNF, HIF-1, and FoxO (Fig. 4g). Anti-CD47 treatment increased the percentage of total CD4^+^ T cells (Fig. 3f), mostly by improving CD4_s1, while treatment of anti-CD47 had little effect on CD4_s2 (Fig. 4d). Anti-CD47 treatment not only changed the percentage of T cells in CD4^+^ T cell clusters but also increased *Pdcd1* expression and decreased *Ctla4* expression (Additional file 1: Figure S3). These data show that anti-CD47 therapy induces a dramatic enhancement in the intratumoral CD4^+^ T cells.

#### Changes in Tregs

Intratumoral Tregs express CD4 and FoxP3 in both human and mouse and play a suppressive role in anti-tumor immunity [29]. Single-cell RNA-seq revealed two Treg clusters (Treg_s1 and Treg_s2) (Fig. 4c). Treg_s2 was distinguishable from Treg_s1 by its higher expression of *Cd4*, *Gzmb*, and *Klrg1*, and lower expression of *Ifit3* (Additional file 1: Figure S3). It is known that immunotherapy like anti-CTLA-4 mAb can decrease the number of intratumoral Tregs in a recent study [30], and in contrast, in our study, the proportion of Tregs increased following anti-CD47 therapy (Fig. 3f). In the mice treated with control mAb, most types of intratumoral Tregs were Treg_s2; however, anti-CD47 treatment induced a shift in the intratumoral Treg cluster toward Treg_s1 (Fig. 4c, d). Furthermore, after anti-CD47 treatment, Tregs displayed transcriptomic changes, including downregulation of *Il10* expression (Additional file 1: Figure S3). In summary, these data indicate that anti-CD47 treatment changes the intratumoral Treg cluster to blunt its inhibitory effect on the tumor response to the treatment.

#### Changes in CD8^+^ T cells

Single-cell RNA-seq revealed eight distinct clusters of CD8^+^ T cells (CD8_s1, CD8_s2, CD8_s3, CD8_s4, CD8_s5, CD8_s6, CD8_s7, and CD8_s8) (Fig. 4c). CD8_s2 selectively expressed *Ly6c2*, *Mx1*, and *Ifit2* (Additional file 1: Figure S3). CD8_s3 selectively expressed *Sell*, *Fam101b*, and *Ccr7*. CD8_s4 selectively expressed *Pim3* and *Ing2*. CD8_s5 selectively expressed *Gzmk* and *Tox*. CD8_s6 selectively expressed *Gzmc*, *Gzmd*, *Prf1*, and *Gzmf*. CD8_s7 selectively expressed *Serpinb1a*, *Il18r1*, and *Cxcr3*. CD8_s8 selectively expressed *Apoe*, *C1qa*, *C1qc*, and *Lgmn*. KEGG revealed that CD8_s8 displayed upregulation in the signaling pathways associated with T cell receptor, PD-1/PD-L1 checkpoint, TNF, and HIF-1 (Fig. 4g). Moreover, both CD8_s1 and CD8_s2 expressed higher levels of *Gzmb*, whereas CD8_s5, CD8_s6, and CD8_s7 expressed higher levels of *Serpinb9* (Additional file 1: Figure S3). *Serpinb9* inhibits the activity of the effector molecule *Gzmb* (granzyme B) [31, 32]. Overexpression of *Serpinb9* may suppress cytotoxic T lymphocytes from eliminating cancer cells. CD8_s1, CD8_s2, and CD8_s3 showed higher gene expression of *Ifit3*, *Ifit3b*, and *Ccl5* (Additional file 1: Figure S3). In contrast, CD8_s3 expressed the lowest levels of *Lag3* and *Pdcd1*. Following anti-CD47 treatment, the number and percentage of CD8_s1, CD8_s2, and CD8_s3 cells increased, whereas the number and proportion of CD8_s4, CD8_s5, CD8_s6, CD8_s7, and CD8_s8 cells decreased (Fig. 4c, e). Anti-CD47 treatment also altered gene expression on a per cell in CD8^+^ T cell clusters, CD8_s1, CD8_s2, and CD8_s3 showed increased expression of *Gzmb*, *Ifitm2*, *Ifit3*, *Tnfrsf1b*, and *Ifng* (Additional file 1: Figure S3). It is known that *Ifng* is produced by activated lymphocytes, it can enhance the anti-tumor effects of the type I interferon [33]. *Ifitm2* is associated with IFN-γ signaling and *Tnfrsf1b* is a member of the TNF-receptor superfamily [34, 35]. Together, these findings demonstrate that anti-CD47 treatment induces a shift in the intratumoral CD8+ T cell cluster toward one that is more activated.

#### Changes in Mki67^high^ cells

Mki67^high^ cells (Mki67_s), a cluster of high expression of the genes linked to cell proliferation, identified by single-cell RNA sequencing (Fig. 4a). Mki67_s contained a mixture of several different subpopulations of immune cells, and the predominant one is CD8^+^ T cells (Fig. 4b and Additional file 1: Figure S3). Mki67_s showed increased expression of *Cd8b1*, *Gzmb*, *Ccl5*, and *Ifit3* (Additional file 1: Figure S3). Anti-CD47 treatment not only enhanced the proportion of Mki67^high^ cells (Fig. 4d), but also altered the gene expression on individual cells because these cells showed increased expression of *Gzmb*, *Ccl5*, and *Ifit3*, and decreased expression of *Lag3* following anti-CD47 treatment (Additional file 1: Figure S3).

### CD47 blockade remodeled intratumoral macrophage compartment

**Fig. 5:**
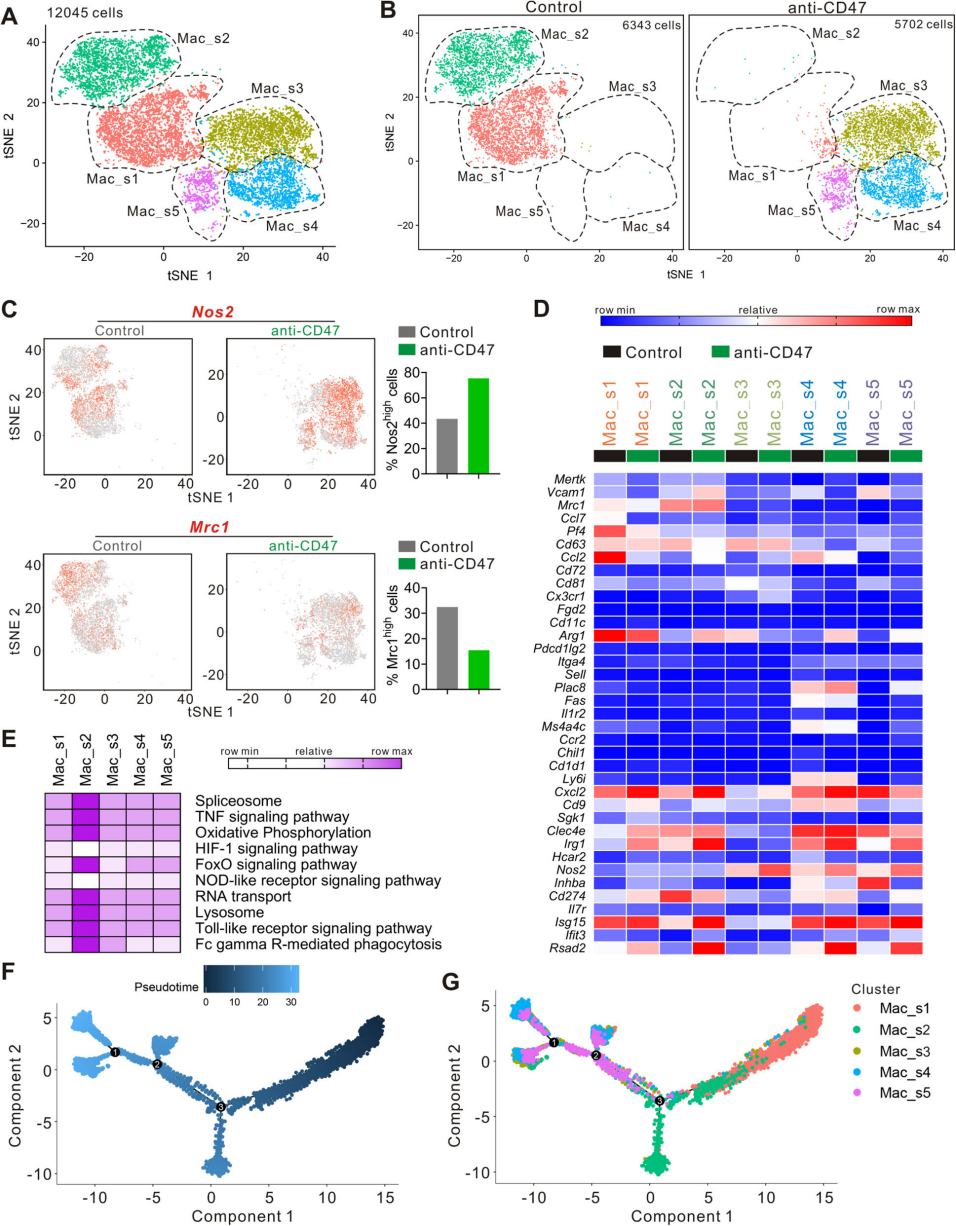
Anti-CD47 treatment remodels intratumoral monocyte/macrophage cells. **a** tSNE plot from merged data of intratumoral monocyte/macrophage cells. **b** tSNE plots with annotated clusters of intratumoral monocyte/macrophage cells. **c** tSNE plot of monocyte/macrophage cells displaying *Nos2* and *Mrc1* expression. **d** Heatmap from scRNA-seq displaying expression of select genes in each monocyte/macrophage cell subpopulation. **e** Heatmap of KEGG identifying pathway enrichment by each subpopulation. **f** tSNE plot with analysis of monocyte/macrophage cells by Monocle2. **g** Monocyte/macrophage subpopulations overlaid on Monocle2 pseudotime plot

Single-cell RNA-seq revealed five distinct clusters of macrophages (Mac_s1, Mac_s2, Mac_s3, Mac_s4, Mac_s5) (Fig. 5a). Following anti-CD47 treatment, the number and proportion of Mac_s1 and Mac_s2 cells were sharply decreased, whereas the number and proportion of Mac_s3, Mac_s4, and Mac_s5 cells were dramatically increased (Fig. 5b). *Nos2* (iNOS), nitric oxide synthase 2, acts as a biologic mediator in antimicrobial and antitumoral activities [36]. *Nos2* is also involved in the regulation of inflammation mainly by increasing the synthesis of pro-inflammatory mediators [37]. In macrophages, *Nos2* plays a key role in tumoricidal action [38]. As shown in Fig. 5c, anti-CD47 treatment induced elevated expression of *Nos2* in intratumoral macrophages, when compared with the control mAb-treated mice. In contrast, *Mrc1* (CD206) involved in anti-inflammatory responses and immunosuppression processes [39]. The intratumoral macrophages of control mAb-treated mice expressed a high level of *Mrc1* gene, whereas the *Mrc1* expression was significantly decreased by anti-CD47 treatment. Immunohistochemical analysis also revealed that targeting CD47 increased the number of iNOS^+^ cells, and reduced CD206^+^ cells (Additional file 1: Figure S2). Mac_s1 cells were characterized by high expression of *Pf4*, *Ccl2*, and *Arg1* (Fig. 5d). Mac_s2 cells were characterized as expressing high levels of *Mrc1* and *Cd274*. Mac_s3 cells expressed the highest level of *Nos2.* Mac_s4 cells selectively expressed *Plac8*, *Fas*, *Ms4a4c*, *Ly6i*, and *Cd9*. KEGG revealed that Mac_s2 displayed upregulation of pathways associated with spliceosome, oxidative phosphorylation, RNA transport, lysosome, and Fc gamma R-mediated phagocytosis and signaling via TNF, FoxO, and toll-like receptor (Fig. 5e).

These data indicated the remodeling of the macrophage compartments induced by anti-CD47 treatment, though it did not shed light on the origin of the cells in each cluster. To further understand the temporal dynamic of macrophage compartment remodeling, Monocle2 was used to analyze these data (Fig. 5f). Mac_s1 cluster might be the start point, and it, towards two distinct fates, becomes the Mac_s2 or the Mac_s4 macrophages (Fig. 5g). This analysis indicated that Mac_s2 could be converted to Mac_s4 or Mac_s5, and anti-CD47 treatment may promote this process.

### Effect of combined targeting of CD47 and PD-L1 in mouse PDAC models

**Fig. 6:**
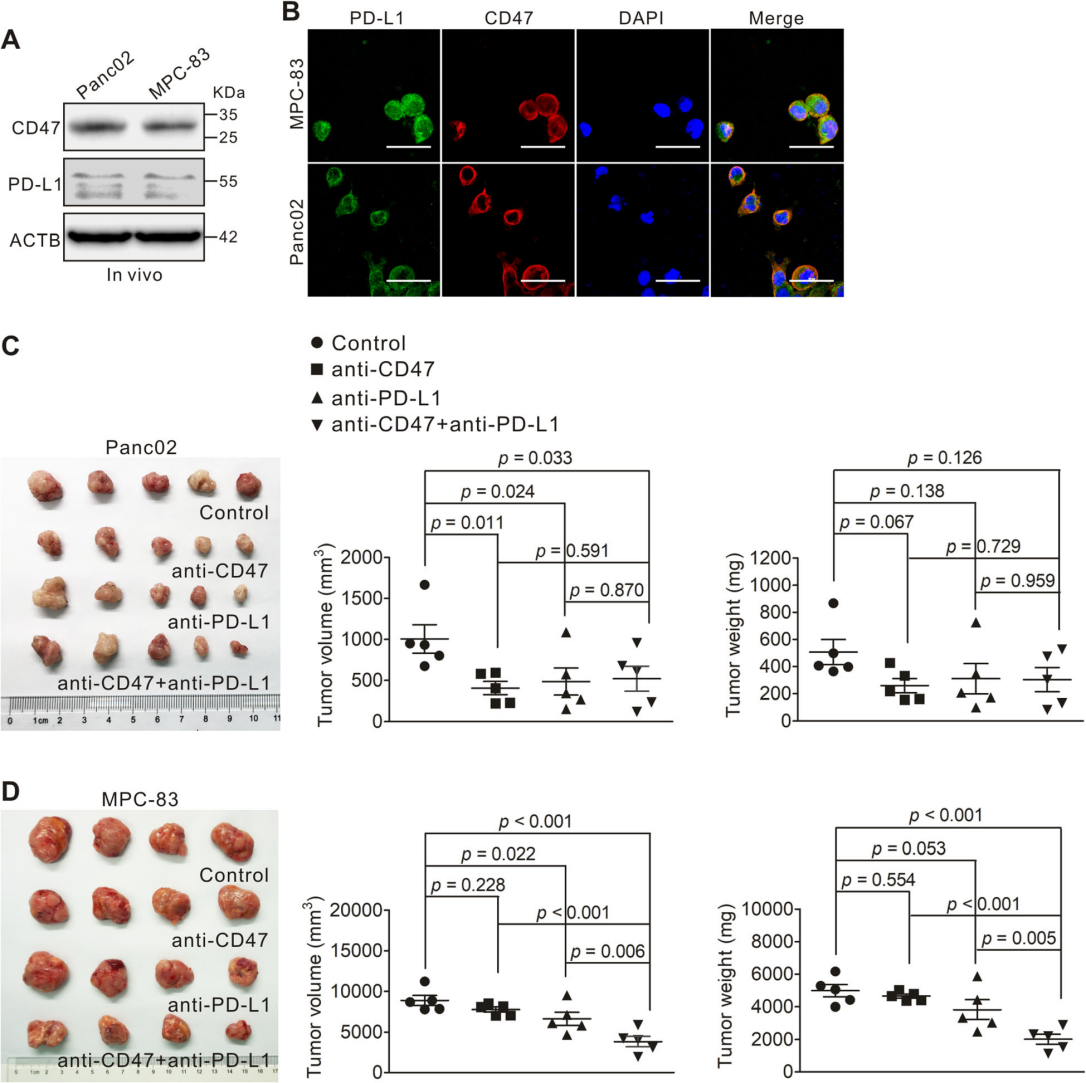
Inhibitory effect of anti-CD47 and anti-PD-L1 targeting on PDAC tumor growth. **a**, **b** Panc02 cells or MPC-83 cells were subcutaneously transplanted into C57BL/6 mice or KM mice to establish pancreatic tumors. CD47 and PD-L1 expression levels on Panc02 and MPC-83 cells from xenografts (in vivo) as measured by immunoblotting (**a**) and immunofluorescence (**b**). **c** Panc02 cells were transplanted to C57BL/6 mice. When the tumor reached 100 mm^3^, tumor-bearing mice were divided into four groups and treated with anti-CD47 and anti-PD-L1 antibodies, either alone or in combination for 14 days. The tumors removed from each group are shown in the left panel; tumor volume (middle panel) and weight (right panel) were compared to those of the untreated control. *p* values were calculated based on a Student’s *t* test. **d** MPC-83 cells were transplanted to KM mice. When the tumor reached 100 mm^3^, tumor-bearing mice were treated as in **c**. The tumors removed from each group are shown in the left panel; tumor volume (middle panel) and weight (right panel) were compared to those of the untreated control. *p* values were calculated based on a Student’s *t* test

To investigate the anti-tumor effect of CD47 and PD-L1 combination treatment, we transplanted Panc02 and MPC-83 cells to C57BL/6 and KM mice. Western blotting and immunoflurescence showed that both Panc02 and MPC-83 cells express CD47 and PD-L1 in vivo (Fig. 6a, b). The tumor-bearing mice were treated with anti-CD47 monoclonal antibody and an anti-PD-L1 mAb, alone or in combination. In the Panc02 tumor model, we found that anti-CD47 mAb or anti-PD-L1 mAb treatment alone resulted in a decreased tumor growth compared to that of untreated animals; however, no synergistic effect was observed (Fig. 6c). For the MPC-83 tumor model, anti-PD-L1 mAb but not anti-CD47 mAb treatment showed significant inhibitory effect on the tumor growth. When both anti-CD47 mAb and anti-PD-L1 mAb were applied, the inhibition of tumor growth was synergistic compared to either anti-CD47 or anti-PD-L1 alone, as assessed by the tumor volume (*p* < 0.001; *p* = 0.006) and weight (*p* < 0.001; *p* = 0.005; Fig. 6d). This result suggests that the efficacy of CD47 blockade or in combination with PD-L1 blockade may depend on the context of TME established by different PDAC tumor cell lines.

**Fig. 7:**
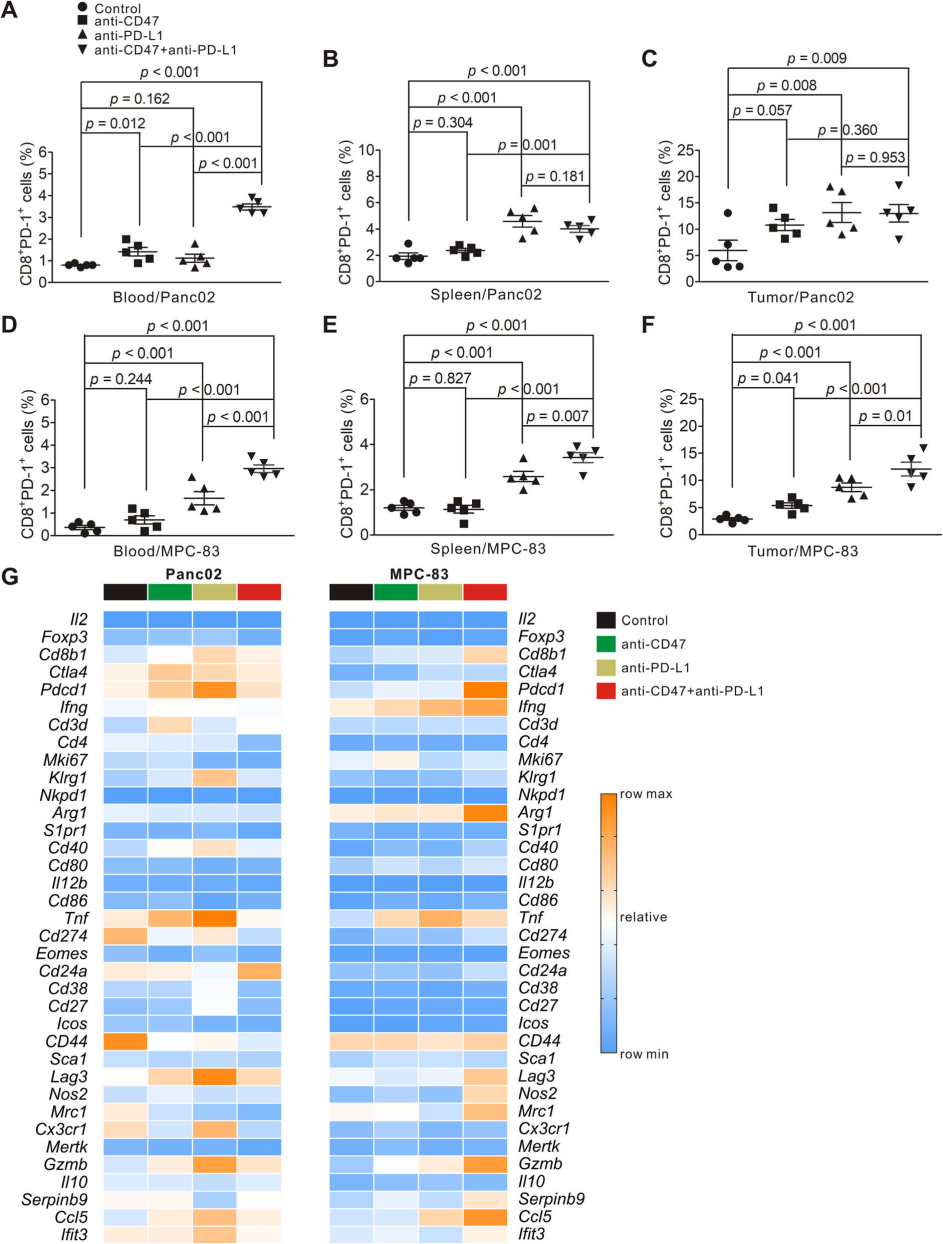
Effect of anti-CD47 and anti-PD-L1 treatment on tumor-infiltrating immune cells in PDAC mouse models. Panc02 or MPC-83 cells were transplanted to C57BL/6 or KM mice. And the tumor-bearing mice were treated as in Fig. 6 c and d. Cell suspensions were prepared from the peripheral blood, spleen, and pancreatic tumor tissues from tumor-bearing mice and analyzed by flow cytometry and bulk RNA-seq. **a**–**c** Quantification of CD8^+^PD-1^+^ T lymphocytes in the Panc02 tumor model in the indicated tissues. **d**–**f** Quantification of CD8^+^ PD-1^+^ T lymphocytes in the MPC-83 tumor model in the indicated tissues. **g** Heatmap from RNA-seq displaying expression of select genes in each tumor-bearing mouse group

To understand the role of CD8^+^ T lymphocytes following targeting of both CD47 and PD-L1, we examined the proportion of PD-1^+^CD8^+^ T cells from lymphocytes isolated from the peripheral blood, spleen, and tumor tissue, respectively, using flow cytometry. An average of 0.8%, 1.94%, and 5.96% CD8^+^ T lymphocytes in the peripheral blood, spleen, and tumor, respectively, from the untreated Panc02 tumor-bearing mice were PD-1 positive (Fig. 7a–c). For MPC-83 tumor-bearing mice, an average of 0.36%, 1.2%, and 2.9% of CD8^+^ T lymphocytes were PD-1 positive in the peripheral blood, spleen, and tumor, respectively (Fig. 7d–f). In both Panc02 and MPC-83 tumor models, we found that anti-CD47 mAb or anti-PD-L1 mAb treatment alone or in combination increased the proportion of PD-1^+^CD8^+^ T lymphocytes in the peripheral blood, spleens, or tumors, when compared to that of untreated animals. Further analysis revealed that the combination treatment increased the proportion of PD-1^+^CD8^+^ T cells in the peripheral blood, when compared to either anti-CD47 or anti-PD-L1 alone in both Panc02 and MPC-83 tumor-bearing mouse models (Fig. 7a, d). In the MPC-83 tumor model, combination treatment increased the proportion of PD-1^+^CD8^+^ T lymphocytes in spleens and tumors compared to either anti-CD47 or anti-PD-L1 alone (Fig. 7e, f). However, this finding was not observed in the Panc02 tumor-bearing mouse model (Fig. 7b, c).

To further understand the functional and transcriptional changes of immune tumor-infiltrating immune cells following combined anti-CD47 and anti-PD-L1 treatment, bulk RNA-seq was performed on the tumor-infiltrating immune cells from each tumor-bearing mouse group. In the Panc02 mouse model, the expression of *Cd8b1*, *Ctla4*, *Pdcd1*, *Lag3*, *Gzmb*, and *Ccl5* were increased by anti-CD47 and anti-PD-L1 immunotherapy alone and in combination, whereas the expression of *Cd274*, *Cd44*, and *Mrc1* was decreased (Fig. 7g). No changes in the expression of other important genes such as *Foxp3*, *Mki67*, *Icos*, *Nos2*, and *Il10* (Fig. 7g). In the MPC-83 mouse model, the expression of *Ifng*, *Tnf*, and *Gzmb* was significantly increased with the treatment of anti-CD47 and anti-PD-L1 mAb either alone or in combination; the expression of *Cd8b1*, *Pdcd1*, *Arg1*, *Lag3*, *Nos2*, *Mrc1*, *Serpinb9*, and *Ifit3* was only increased by the combination treatment (Fig. 7g). These data demonstrated that the effect of targeting CD47 and PD-L1 on the tumor-infiltrating immune cells in mouse PDAC models is indeed dependent on the TME established by different PDAC cell lines.

## Discussion

Macrophages are some of the key tumor-infiltrating immune cells in the TME of PDAC [7, 9]. Our data show that the number of tumor-infiltrating CD68^+^ M correlated with that of tumor-infiltrating CD163^+^ M2 and with the tumor expression of CD47, while no significant correlation was found between CD47 expression and the proportion of tumor-infiltrating CD163^+^ M2. Importantly, tumor-infiltrating CD68^+^ M and tumor expression of CD47 correlated with clinico-pathological features of PDAC patients in our study. However, high CD163^+^ M2 infiltrates correlated with high pT-stage and large tumor diameter, suggesting that M2 macrophages may contribute to the tumor growth and progression of PDAC. Our data demonstrates that macrophages of the TME play key roles in the outcomes of patients with PDAC.

We further show that PDAC patients with high tumor expression of CD47 and high tumor-infiltrating macrophages were associated with poor clinical outcomes. When CD47, PD-L1, CD68^+^ M, and CD163^+^ M2 were paired for survival analysis, three patient groups (CD47^high^ /PD-L1^high^, CD47^high^/CD68^+^ M^high^, and CD47^high^/CD163^+^ M2^high^) were associated with shorter OS, while two groups (CD47^low^/CD68^+^ M^low^ and CD47^low^/CD163^+^ M2^low^) were associated with longer OS. To our knowledge, this is the first study to show the prognostic value of CD47 and its correlation with tumor-infiltrating macrophages in PDAC. This is important because it demonstrates that the composition of the infiltrating immune cells inside the TME compartment may be prognostic of survival in patients with PDAC.

Several previous studies showed that anti-CD47 targeting was effective in suppressing tumor growth in some human cancer xenograft models [27, 28, 40, 41]. Michaels et al. [18] reported that liver macrophages reduced the progression of PDAC micro-metastasis. In our study, we did not observe significantly different tumor burden between nude mice and NCG mice in our PDX models. Surprisingly, anti-CD47 treatment had a limited anti-tumor effect in PDX models, though tumor-bearing nude mice showed less tumor burden when treated with anti-CD47. This limitation may be due to the fact that CD47 mediates T-cell function that was missing in the NCG mouse [42, 43].

Using syngeneic mouse models implanted with PDAC cells, we observed that CD47 blockade alone inhibited tumor growth in the Panc02, but not MPC-83 syngeneic mouse model, though in both models, there were increased tumor-infiltrating PD-1^+^CD8^+^ T cells. Our unbiased single-cell RNA-seq data using the immune cells of both mouse models revealed that the intratumoral lymphocytes and macrophages were dramatically remodeled by anti-CD47 treatment. Single-cell RNA-seq provides a unique advantage compared to the unsupervised analysis of cell subpopulations, with an ability to analyze thousands of genes on an individual cell [44, 45]. Our study demonstrated that anti-CD47 treatment led to changes in the tumor microenvironment with increased pro-inflammatory macrophages that exhibit anti-tumor effect, while reduced anti-inflammatory macrophages that are associated with immunosuppression. Moreover, anti-CD47 treatment increased the proportions and numbers of intratumoral lymphoid cells. These results indicate that both innate and adaptive immunity are important in mediating the anti-CD47 immunotherapeutic effect.

Our flow cytometry data revealed that combination treatment with anti-CD47 and anti-PD-L1 increased the levels of tumor PD-1^+^CD8^+^ T cells infiltrate and decreased the tumor burden in the MPC-83 but not in the Panc02 syngeneic mouse model. The mechanism of such differential effect in different mouse syngeneic models of PDAC is intriguing [42, 43, 46] as our RNA-seq data revealed enhanced expressions of several key immune-activating genes including *Pdcd1*, *Arg1*, *Nos2*, *Gzmb*, and *Ifit3* in MPC-83 mouse model but not in Panc02 model. The exact mechanism of such differential effect to the anti-CD47 and anti-PD-L1 combined blockade requires further investigation.

## Conclusions

In summary, our study has shown that the tumor expression of CD47 correlated with the levels of tumor-infiltrating macrophages and may serve as an independent prognostic marker in patients with PDAC. CD47 targeting remodels the TME of PDAC and altered the infiltrating immune cell composition. Combination targeting of both CD47 and PD-L1 resulted in synergistic inhibitory effect on tumor growth in the MPC-83 but not Panc02 syngeneic PDAC mouse model due to their differential effect on the key immune-activating genes and infiltrating immune cells in the TME. Further elucidating of this intriguing differential effect of combined anti-CD47 and anti-PD-L1 blockade on the PDAC mouse models established from different PDAC cell line may yield further insight on the regulation of TME by immunotherapy in patients with PDAC.

## Supplementary information

**Additional file 1: Table S1.** Clinical and pathologic features of PDAC patients. **Table S2**. Univariate and multivariate Cox proportional analysis for overall survival. **Table S3.** Multivariate analysis of CD47 expression combined with PD-L1 expression or TAM in PDAC patients. Analysis was adjusted for diameter, grade, and TNM stage. **Figure S1.** Tumor-infiltrating CD45^+^ immune cells in Panc02 tumor-bearing mice for single-cell analysis. **Figure S2.** Immunostaining of CD4, CD8, iNOS, and CD206 in Panc02 tumors. **Figure S3.** Heatmap from scRNA-seq displaying expression of select genes in each lymphoid cell subpopulation.

## Data Availability

Please contact the author for data requests.

## Abbreviations

CD163+ M2: CD163+ M2 macrophages
CD68+ M: CD68+ pan-macrophages
FBS: Fetal bovine serum
HR: Hazard ratio
IHC: Immunohistochemistry
IL-10: Interleukin-10
mAb: Monoclonal antibody
OS: Overall survival
PBMCs: Peripheral blood mononuclear cells
PDAC: Pancreatic ductal adenocarcinoma
PDX: Patient-derived xenograft
SD: Standard deviation
SIRPα: Signal regulatory protein α
TAM: Tumor-associated macrophages
TME: Tumor microenvironment
