# Supplementary material for: Single-cell RNA sequencing reveals compartmental remodeling of tumor-infiltrating immune cells induced by anti-CD47 targeting in pancreatic cancer
Source: J Hematol Oncol. 2019 Nov 27;12:124. doi: 10.1186/s13045-019-0822-6 (PMC6880569; doi:10.1186/s13045-019-0822-6)
Supplement: Supplementary file 1 — Additional file 1: Table S1. Clinical and pathologic features of PDAC patients. Table S2. Univariate and multivariate Cox proportional analysis for overall survival. Table S3. Multivariate analysis of CD47 expression combined with PD-L1 expression or TAM in PDAC patients. Analysis was adjusted for diameter, grade, and TNM stage. Figure S1. Tumor-infiltrating CD45+ immune cells in Panc02 tumor-bearing mice for single-cell analysis. Figure S2. Immunostaining of CD4, CD8, iNOS, and CD206 in Panc02 tumors. Figure S3. Heatmap from scRNA-seq displaying expression of select genes in each lymphoid cell subpopulation. [file 13045_2019_822_MOESM1_ESM.docx]

**Single-cell RNA sequencing reveals compartmental remodeling of tumor-infiltrating immune cells induced by anti-CD47 targeting in pancreatic cancer**

Yu Pan^1#^, Fengchun Lu^1#^, Qinglin Fei^1^, Xingxing Yu^1^, Ping Xiong^2^, Xunbin Yu^3^, Yuan Dang^4^, Zelin Hou^1^, Wenji Lin^5^, Xianchao Lin^1^, Zheyang Zhang^6^, Minggui Pan^7^, Heguang Huang^1*^

**Supplementary Materials**

**(Table S1 – S3 and Figure S1 – S3)**

| **Table S1** Clinical and pathologic features of PDAC patients | | | | |  |  |  |  |  |  |
| --- | --- | --- | --- | --- | --- | --- | --- | --- | --- | --- |
|  | n = 106 | Expression of CD47 | | *P*-Value | CD68+ M | | *P*-Value | CD163+ M2 | | *P*-Value |
|  |  | High | Low |  | High | Low |  | High | Low |  |
| Gender |  |  |  |  |  |  |  |  |  |  |
| Male | 62 (58.5%) | 37 | 25 | 0.68 | 31 | 31 | 0.644 | 29 | 33 | 0.285 |
| Female | 44 (41.5%) | 28 | 16 |  | 20 | 24 |  | 16 | 28 |  |
| Age (years) |  |  |  |  |  |  |  |  |  |  |
| >=65 | 42 (39.6%) | 26 | 16 | 0.92 | 19 | 23 | 0.631 | 20 | 22 | 0.383 |
| <65 | 64 (60.4%) | 39 | 25 |  | 32 | 32 |  | 25 | 39 |  |
| Tumor site |  |  |  |  |  |  |  |  |  |  |
| Head | 65 (61.3%) | 42 | 23 | 0.381 | 31 | 34 | 0.913 | 30 | 35 | 0.332 |
| Other | 41 (38.7%) | 23 | 18 |  | 20 | 21 |  | 15 | 26 |  |
| Diameter (cm) |  |  |  |  |  |  |  |  |  |  |
| <4 | 49 (46.2%) | 30 | 19 | 0.985 | 21 | 28 | 0.315 | 16 | 33 | **0.058** |
| >=4 | 57 (53.8%) | 35 | 22 |  | 30 | 27 |  | 29 | 28 |  |
| CA 19-9 (U/ml) |  |  |  |  |  |  |  |  |  |  |
| <37 | 27 (25.5%) | 18 | 9 | 0.509 | 15 | 12 | 0.37 | 12 | 15 | 0.808 |
| >=37 | 79 (74.5%) | 47 | 32 |  | 36 | 43 |  | 33 | 46 |  |
| Procedure |  |  |  |  |  |  |  |  |  |  |
| PD | 58 (56.3%) | 38 | 20 | 0.329 | 26 | 32 | 0.457 | 25 | 33 | 0.266 |
| DP | 38 (33.3%) | 21 | 17 |  | 20 | 18 |  | 26 | 24 |  |
| TP | 10 (10.4%) | 6 | 4 |  | 5 | 5 |  | 6 | 4 |  |
| pT-staging |  |  |  |  |  |  |  |  |  |  |
| pT1 | 3 (2.3%) | 3 | 0 | 0.545 | 2 | 1 | 0.089 | 2 | 1 | **0.015** |
| pT2 | 37 (41.4%) | 23 | 14 |  | 13 | 24 |  | 9 | 28 |  |
| pT3 | 46 (42.5%) | 28 | 18 |  | 24 | 22 |  | 24 | 22 |  |
| pT4 | 20 (13.8%) | 11 | 9 |  | 12 | 8 |  | 10 | 10 |  |
| pN-staging |  |  |  |  |  |  |  |  |  |  |
| pN0 | 40 (37.7%) | 21 | 19 | 0.147 | 17 | 23 | 0.368 | 14 | 26 | 0.227 |
| pN+ | 66 (62.3%) | 44 | 22 |  | 34 | 32 |  | 31 | 35 |  |
| pM-staging |  |  |  |  |  |  |  |  |  |  |
| pM0 | 101 | 62 | 39 | 0.951 | 48 | 53 |  | 43 | 58 | 0.909 |
| pM1 | 5 | 3 | 2 |  | 3 | 2 |  | 2 | 3 |  |
| Grading |  |  |  |  |  |  |  |  |  |  |
| G1 | 5 (4.7%) | 3 | 2 | 0.27 | 2 | 3 | 0.247 | 1 | 4 | 0.421 |
| G2 | 79 (74.5%) | 46 | 33 |  | 36 | 43 |  | 33 | 46 |  |
| G3 | 21 (19.8%) | 15 | 6 |  | 12 | 9 |  | 10 | 11 |  |
| G4 | 1 (1.0%) | 1 | 0 |  | 1 | 0 |  | 1 | 0 |  |
| Vascular invasion |  |  |  |  |  |  |  |  |  |  |
| Yes | 31 (29.2%) | 15 | 16 | 0.079 | 15 | 16 | 0.971 | 15 | 16 | 0.427 |
| No | 75 (70.8%) | 50 | 25 |  | 36 | 39 |  | 30 | 45 |  |
| Chemotherapy |  |  |  |  |  |  |  |  |  |  |
| Yes | 54 (50.9%) | 31 | 23 | 0.399 | 25 | 29 | 0.703 | 21 | 33 | 0.878 |
| No | 52 (49.1%) | 34 | 18 |  | 26 | 26 |  | 24 | 28 |  |
| CD68+ M |  |  |  |  |  |  |  |  |  |  |
| High | 51 (48.1%) | 38 | 13 | **0.007** | — | — | — | — | — | — |
| Low | 55 (51.9%) | 27 | 28 |  | — | — |  | — | — |  |
| CD163+ M2 |  |  |  |  |  |  |  |  |  |  |
| High | 45 (42.5%) | 29 | 16 | 0.571 | 38 | 7 | **< 0.001** | — | — | — |
| Low | 61 (57.5%) | 36 | 25 |  | 13 | 48 |  | — | — |  |
| Abbreviations: M, macrophages; M2, M2 macrophages; PD, pancreaticoduodenectomy; | | | | | | | | | | |
| DP, distal pancreatectomy; TP, total pancreatectomy | | | | | | | | | | |

| **Table S2**. Univariate and multivariate Cox proportional analysis for overall survival | | | | | | | | |
| --- | --- | --- | --- | --- | --- | --- | --- | --- |
| Variable | n | Univariate analysis | | |  | Multivariate analysis | | |
|  |  | HR | 95% CI | *P*-value |  | HR | 95% CI | *P*-value |
| Gender |  |  |  |  |  |  |  |  |
| Male | 62 | 1 |  |  |  |  |  |  |
| Female | 44 | 0.863 | 0.535-1.392 | 0.545 |  |  |  |  |
| Age (years) |  |  |  |  |  |  |  |  |
| >=65 | 42 | 1 |  |  |  |  |  |  |
| <65 | 64 | 0.998 | 0.976-1.025 | 0.996 |  |  |  |  |
| Tumor site |  |  |  |  |  |  |  |  |
| Head | 65 | 1 |  |  |  |  |  |  |
| Other | 41 | 0.631 | 0.388-0.992 | 0.056 |  |  |  |  |
| Diameter (cm) |  |  |  |  |  |  |  |  |
| <4 | 49 | 1 |  |  |  | 1 |  |  |
| >=4 | 57 | 1.643 | 1.027-2.626 | **0.038** |  | 1.626 | 1.006-2.626 | **0.047** |
| CA 19-9 (U/ml) |  |  |  |  |  |  |  |  |
| <37 | 27 | 1 |  |  |  |  |  |  |
| >=37 | 79 | 0.993 | 0.591-1.669 | 0.98 |  |  |  |  |
| pT-staging |  |  |  |  |  |  |  |  |
| pT1+pT2 | 40 | 1 |  |  |  |  |  |  |
| pT3+pT4 | 66 | 1.268 | 0.957-1.680 | 0.098 |  |  |  |  |
| pN-staging |  |  |  |  |  |  |  |  |
| pN0 | 40 | 1 |  |  |  | 1 |  |  |
| pN+ | 66 | 1.82 | 1.365-2.427 | **< 0.001** |  | 1.831 | 1.339-2.506 | **< 0.001** |
| pM-staging |  |  |  |  |  |  |  |  |
| pM0 | 101 |  |  |  |  |  |  |  |
| pM1^a^ | 5 | — | — | — |  | — | — | — |
| Grading |  |  |  |  |  |  |  |  |
| G1^a^ | 5 | — | — | — |  | — | — | — |
| G2 | 79 | 1 |  |  |  | 1 |  |  |
| G3 | 21 | 2.478 | 1.438-4.269 | **0.001** |  | 1.745 | 1.137-2.680 | **0.011** |
| G4^a^ | 1 | — | — | — |  | — | — | — |
| Vascular invasion |  |  |  |  |  |  |  |  |
| No | 31 | 1 |  |  |  |  |  |  |
| Yes | 75 | 1.024 | 0.618-1.696 | 0.928 |  |  |  |  |
| Chemotherapy |  |  |  |  |  |  |  |  |
| No | 54 | 1 |  |  |  |  |  |  |
| Yes | 52 | 0.71 | 0.445-1.134 | 0.152 |  |  |  |  |
| CD47 expression |  |  |  |  |  |  |  |  |
| Low | 65 | 1 |  |  |  | 1 |  |  |
| High | 41 | 1.673 | 1.030-2.716 | **0.037** |  | 1.703 | 1.031-2.815 | **0.038** |
| CD68+ M |  |  |  |  |  |  |  |  |
| Low | 51 | 1 |  |  |  | 1 |  |  |
| High | 55 | 1.892 | 1.170-3.059 | **0.009** |  | 1.853 | 1.106-3.106 | **0.012** |
| CD163+ M2 |  |  |  |  |  |  |  |  |
| Low | 45 | 1 |  |  |  | 1 |  |  |
| High | 61 | 1.845 | 1.143-2.979 | **0.012** |  | 1.898 | 1.139-3.165 | **0.014** |
| Abbreviations: HR, hazard ratio; CI, confidence interval; | | | | | | | | |
| ^a^ Survival analysis not performed due to small number of cases   \| **Table S3.** Multivariate analysis of CD47 expression combined with PD-L1 expression or TAM in PDAC patients. Analysis was adjusted for diameter, grade, and TNM stage \| \| \| \| \| --- \| --- \| --- \| --- \| \| Biomarkers \| HR \| 95% CI \| *P*-value \| \| OS \|  \|  \|  \| \| CD47^high^ / PD-L1^high^ (no/yes) \| 1.71 \| 0.850-3.438 \| 0.132 \| \| CD47^high^ / CD68^+^ M^high^ (no/yes) \| 2.126 \| 1.244-3.634 \| **0.006** \| \| CD47^high^ / CD163^+^ M2^high^ (no/yes) \| 1.873 \| 1.044-3.359 \| **0.035** \| \| CD47^low^ / CD68^+^ M^low^ (no/yes) \| 0.47 \| 0.264-0.836 \| **0.01** \| \| CD47^low^ / CD163^+^ M2^low^ (no/yes) \| 0.376 \| 0.202-0.700 \| **0.002** \| | | | | | | | | |


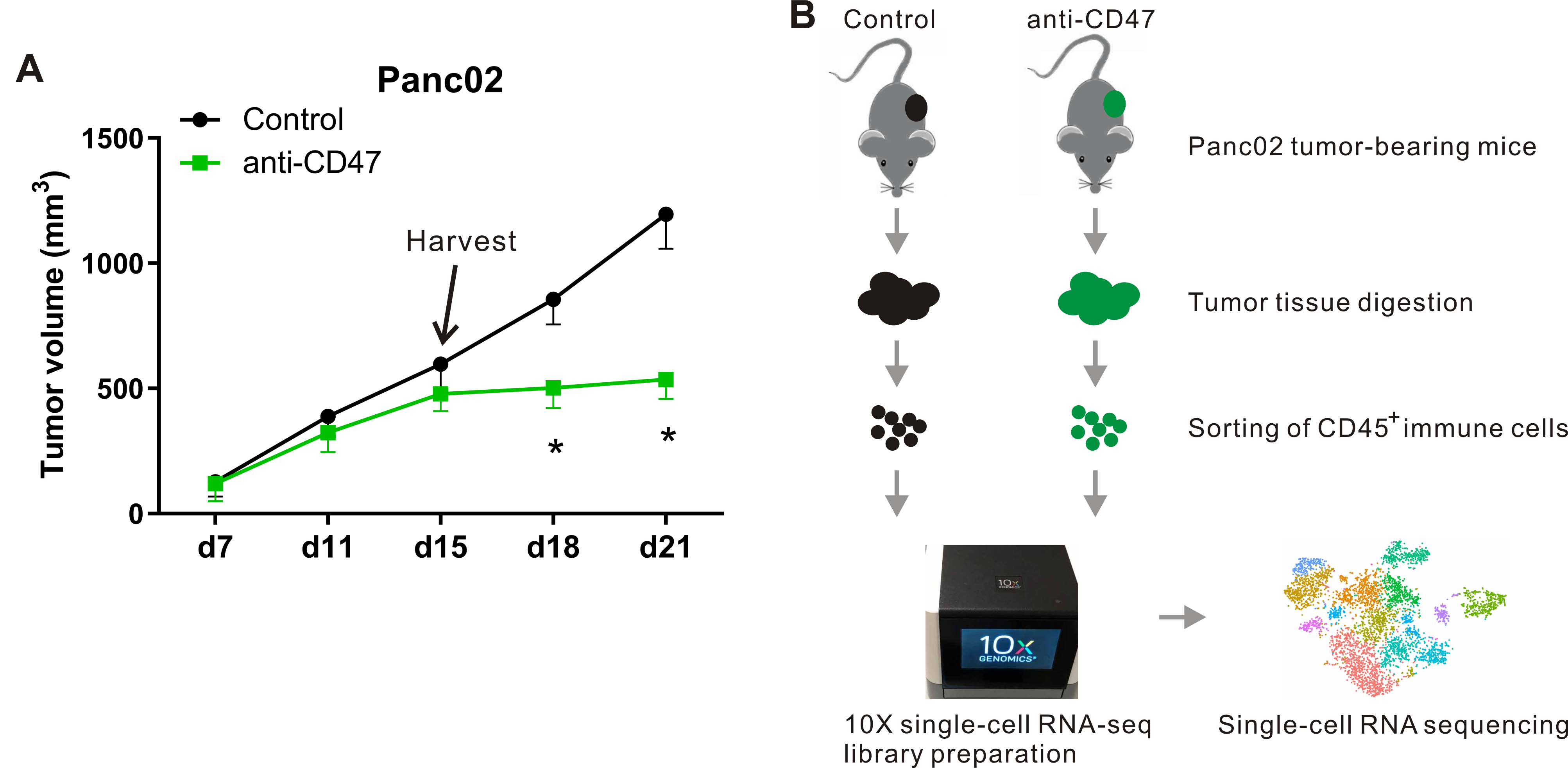


**Figure S1.** Tumor-infiltrating CD45^+^ immune cells in Panc02 tumor-bearing mice for single-cell analysis. (**A**) Panc02 tumor growth in mice treated with control or anti-CD47 mAb. (**B**) Experimental schematic. Panc02 cells were subcutaneously implanted into 10 C57BL/6 mice. When the tumor reached 100 mm^3^, tumor-bearing mice were randomly divided into two groups. Then, tumor-bearing mice were treated with mouse IgG (200 μg/day i.p.), or an anti-mouse CD47 in vivo mAb (200 μg/day i.p.). Tumors were harvested on day 15 after tumor transplant, digested and stained for CD45 and sorted. CD45^+^ immune cells were subjected to scRNA-seq by using the 10x Genomics pipeline. Immune cells from 5 mice from control or anti-CD47 group were pooled before sorting.

**Figure S2.** Immunostaining of CD4, CD8, iNOS, and CD206 in Panc02 tumors. (**A**) Staining with an anti-CD4, anti-CD8, anti-iNOS, and anti-CD206 antibody in tumor tissue samples of Panc02 tumor-bearing mice. Low magnification (100×) and high magnification (200×) images were obtained. Scale bar = 100 µm (red line at the bottom left). (**B**) Positive cells (brown) were analyzed by performing a Student’s t-test. Data represent the mean ± SD. ^*^P < 0.05 when compared with the control group.


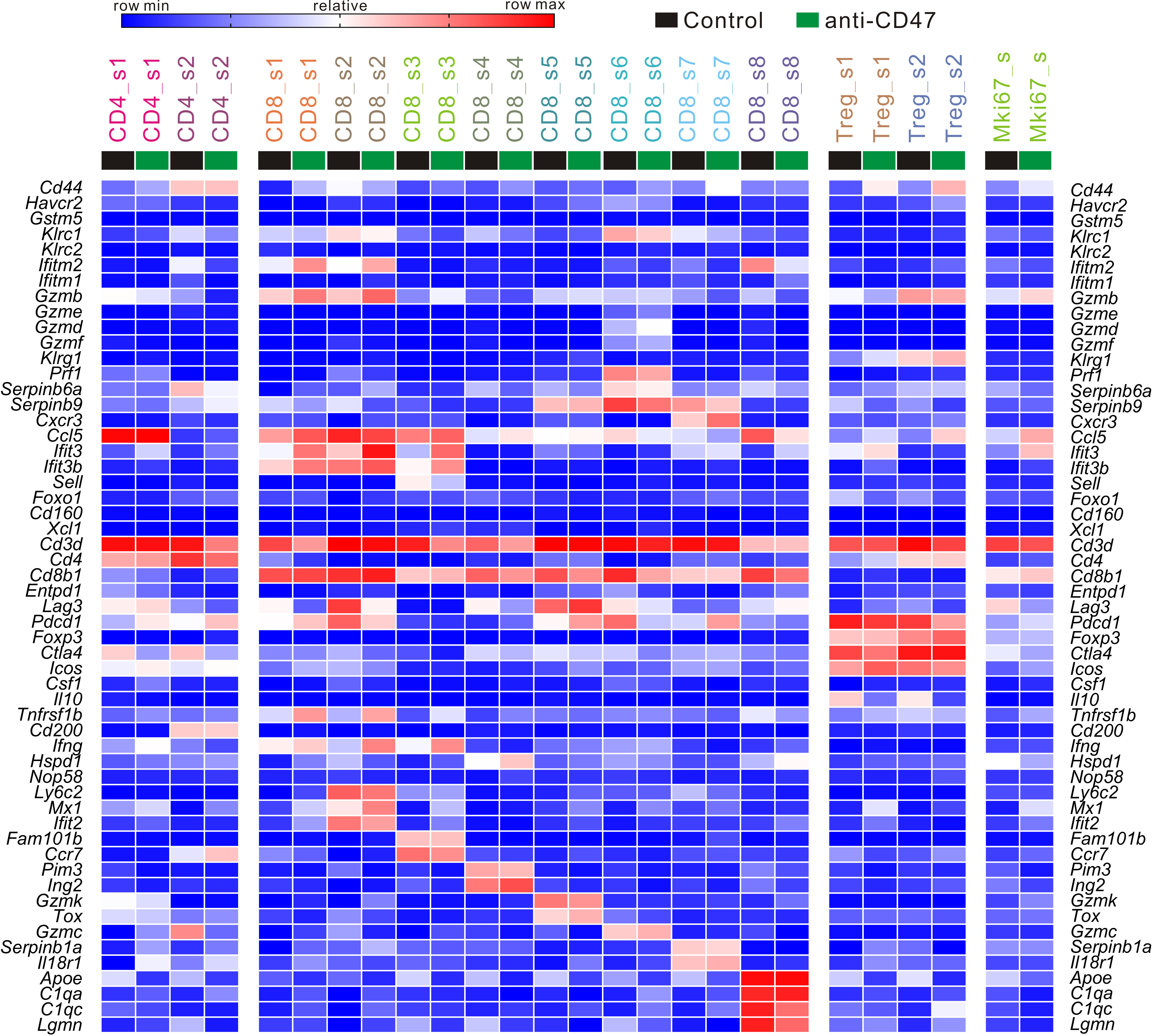


**Figure S3.** Heatmap from scRNA-seq displaying expression of select genes in each lymphoid cell subpopulation.
